# Implementation of a Teaching Electronic Medical Record within Didactic Instruction Using a Drug Information Question Assignment

**DOI:** 10.3390/pharmacy9010035

**Published:** 2021-02-11

**Authors:** Jacqueline Wasynczuk, Amy H. Sheehan

**Affiliations:** 1 Department of Pharmacy Practice, Jefferson College of Pharmacy, Thomas Jefferson University, Philadelphia, PA 19107, USA; 2 Department of Pharmacy Practice, Purdue University College of Pharmacy, Purdue University, West Lafayette, IN 47907, USA; hecka@purdue.edu

**Keywords:** pharmacy education, electronic medical records, health informatics, drug information

## Abstract

Background: Pharmacy graduates are expected to be practice-ready to deliver quality patient care, which includes having comprehensive knowledge of health informatics and electronic medical records (EMRs). The purpose of this study was to (1) incorporate an EMR within a pharmacy student assignment, and (2) assess student perceptions of use of the EMR. Methods: Student pharmacists received a patient-specific drug-related question and were required to use an EMR to provide an accurate response. Following completion of the assignment, students were invited to complete a retrospective, pre-post survey instrument to collect their perceptions. Results: Only 28.8% of respondents reported prior experience using an EMR. Student perceptions about use of an EMR within the didactic setting significantly improved from before to after the assignment. Differences were found in respondents who agreed that didactic use of an EMR increased their confidence in obtaining information from an EMR (20.5% to 82.8%) and improved their knowledge of EMR systems (61.4% to 89.3%). Conclusions: Implementation of an EMR within didactic instruction may serve as the first exposure to health informatics for students and positively impacts student perceptions of these tools prior to entry into pharmacy practice.

## 1. Introduction

Within the United States, the Accreditation Council for Pharmacy Education (ACPE) 2016 Standards include health informatics as a required element, with a clear expectation that graduating students are ‘practice ready’ and able to apply comprehensive knowledge to the delivery of quality patient care in a variety of entry-level practice settings [1]. The importance of didactic training in health informatics has been emphasized by the increased prevalence of electronic health record (EHR) systems in institutions nationwide. This is a result of the Health Information Technology for Economic and Clinical Health Act of 2009, which provides reimbursements to healthcare institutions for meeting standards of meaningful use [2]. The Centers for Medicare & Medicaid Services established Medicare and Medicaid EHR Incentive Programs, now known as Promoting Interoperability programs, to encourage professionals, eligible hospitals, and critical access hospitals to demonstrate meaningful use of certified EHR technology (CEHRT) [3,4]. As of 2015, 96% of non-federal acute care hospitals possessed CEHRT [5]. 

Access to accurate and up-to-date patient information is paramount for the pharmacist to practice patient-centered care and optimize therapeutic outcomes as collection of patient information is the first step to evidence-based pharmacy practice [6]. EHR systems provide the primary source for access to valuable patient-specific information such as the current medication list, medication use history, medical history, current laboratory and clinical findings, as well as relevant social, economic, and behavioral information [7]. Therefore, it is apparent that student pharmacists should be knowledgeable and confident with utilization of electronic health record systems, even prior to their introductory pharmacy practice experiences. 

Previous studies have shown improvement in student pharmacists’ knowledge, perceptions, and confidence in health informatics when the utilization of an electronic medical record (EMR), a type of EHR of patients’ medical charts within a specific institution, is incorporated as part of didactic training [8,9,10,11]. This integration has included laboratory sessions in which student pharmacists were required to complete specific tasks within the EMR and lectures utilizing a team-based learning approach. For example, Kirwin et al. incorporated an EMR within a pharmaceutical care skills laboratory course. Students within this course were to identify drug-therapy problems and recommend solutions with patient specific cases. This study found an overall improvement in student confidence based on survey responses [10]. In another study, by Wisniewski et al., an EHR was used within an order verification exercise in conjunction with a drug information question telephone call exercise in a laboratory course. This study had focused the use of EHR for the order verification activity and had found that students performed well on both the order verification and drug information call exercise [12].

Despite the improvement of student confidence in utilizing an EMR within didactic instruction, a survey conducted of schools of pharmacy in 2015 estimated that only 37% of pharmacy schools use some type of teaching EHR [13]. In another study conducted in 2015, it was found that only 36% of schools of pharmacy curricula include a stand-alone pharmacy informatics course [14]. These and additional previous studies had identified that while health informatics is commonly integrated within pharmacy courses, incorporation of an EHR or EMR as part of an asynchronous drug information (DI) assignment outside of a laboratory course has yet to be evaluated [12,13,14,15,16]. 

Purdue University College of Pharmacy (PUCOP) implemented use of a teaching EMR in August 2017. Its use in fall of 2017 was mainly within the pharmacy program laboratory courses. Given the importance of patient-centered care, incorporation of the EMR within the required DI and literature evaluation course appeared to be a novel way to emphasize the importance of patient-specific background information when answering drug-related requests from health care professionals. A learning objective of the PUCOP’s DI and literature evaluation course was for students to be able to demonstrate the ability to incorporate DI, patient-specific factors, and other pertinent information in the delivery of pharmaceutical care. The DI question assignment was designed to assess this course objective in the context of answering a clinical question. 

Given this was the first semester for PUCOP to utilize an EMR and that, to our knowledge, this was also the first implementation of an EMR for an assignment outside of a laboratory course, the focus of analysis was student perceptions of its use. In support of analysis of student perceptions are theories about motivation in which confidence may influence a person’s learning [17]. In goal-oriented theories, learners with low confidence may demonstrate low performance [18]. In the self-determination theory, the perceived ability of a person plays a role in their competence and subsequently their motivation [17,19]. Therefore, the objectives of this project were to: (1) incorporate use of a teaching EMR within a DI assignment to emphasize the importance of patient-centered care and (2) assess student perceptions of EMR use in the didactic setting.

## 2. Materials and Methods 

### 2.1. Research Setting 

The EMR adopted by the PUCOP is a web-based platform that includes medical records of real patients from historic hospital records. These records had been de-identified and integrated into the platform, allowing student pharmacists to access patient lists and view patient information including medication orders, vital signs, and laboratory values. Student pharmacists could also view and write assessment notes within a patient profile using the teaching EMR. The EMR platform used had been a previous system used at a healthcare institution. The patient data within this system is from real-patient data that has been anonymized for use within an educational setting.

During the 2017 fall semester, the teaching EMR was incorporated within the required DI and literature evaluation course with the goal of highlighting the importance of providing drug-related information that is specific to the patient. In line with ACPE’s 2016 required standard of health information retrieval and evaluation [1], this second-professional course is designed to provide student pharmacists the ability to effectively search medical literature, critically analyze and apply pertinent drug and disease information within the delivery of patient-centered care. This required three-credit hour course takes place over 12 weeks, with 2 two-hour class meetings each week, in which a combination of lecture and active-learning discussion activities occur. Pre-requisites to this course included an introduction to statistical methods and introductory pharmacy skills labs. Patient-specific DI questions are assigned to each student as part of the first written assignment. Additional assessments in the course included quizzes, journal club critiques, a drug monograph assignment, class participation, and a final exam. The DI question assignment has been an integral part of the drug information course at PUCOP for over 15 years and consists of two parts: (1) gathering pertinent patient-specific background information (worth 24 points); and (2) providing a written response (worth 36 points) for a total of 60 points out of the 550 total course points for the semester (approximately 10.9% of the course grade). Within the third week of the course, students receive their assigned DI question verbally during an initial phone call with an instructor playing the role of a prescriber with a drug-related question. Prior class meetings within the course had taught on drug information resources, literature search strategies, and providing patient-specific drug information. The phone call takes place outside of class at an agreed upon time between each instructor and student during the course of one day. The instructor has all information regarding the details of the patient case and the reason the question is being asked. However, instructors only provide specific details if the student specifically asks for the information. Students would receive one of seven potential questions from one of 20 possible instructors. Multiple instructors, including additional faculty and fourth-year student pharmacists affiliated with the PUCOP, are necessary in order to deliver mock DI question phone calls to approximately 150 students within a one-day period. Students are graded for competency in professionalism, obtaining all relevant patient-specific information, determining that the question was patient specific, obtaining information about the requestor, and for understanding of the context of the question using a standard rubric. Understanding the context of the question is necessary for students to be able to provide an appropriate and accurate patient-centered clinical recommendation and answer, without which, responses to the question could result in patient harm. Prior to implementation of the teaching EMR, the only source of patient case information for the drug information question was via the assigned instructor. If students failed to collect patient-specific details during the initial phone call, they would need to follow-up with the instructor via e-mail.

### 2.2. Implementation of Teaching EMR

During the fall semester of 2017, the drug information question assignment was developed using patient cases readily available within the teaching EMR. As in the past, students obtained initial patient information (e.g., patient name, medical record number, primary diagnosis) verbally during an initial phone call with an instructor playing the role of a prescriber with a drug-related question. However, to more adequately mimic real practice, after the phone call each student could then use the EMR to obtain more information about the patient. Typically, this ranged from four to five patient-specific factors relevant to the DI question. Information obtained via the EMR included drug allergies, concomitant medications, comorbidities, vital signs, renal and liver function, office visit notes, and other relevant laboratory findings. For this first part of the assignment, students were graded for competency using the same rubric that was used in previous years. All questions asked for a medication dosing recommendation, usually in regard to an off-label use, for a specific patient and required the students to obtain additional information to fully, and accurately understand the context of the question. An example of a DI question asked by instructors can be found in Appendix A. 

The second part of the assignment was not altered from previous years and students were required to submit a patient-specific written response that answered the drug-related question one week later. However, access to the EMR could be obtained at any time via a web address link with an institution specific login anywhere the student had internet access. Students also had access to all of the institution’s library resources, including several drug databases and literature search engines. Prior to this assignment, students received basic training on how to use the teaching EMR, which included successful completion of an EMR competency quiz within the pharmacy program laboratory course. At this point, students had not used the EMR for previous or concurrent courses. The written portion of the assignment was graded for competency in providing an introduction with the context of the question; providing relevant information from primary and tertiary literature; appropriately identifying patient-specific issues related to the question; using appropriate referencing, grammar, and spelling; and providing an accurate and complete conclusion and recommendation based on the literature and patient-specific factors. Without the patient-specific factors, the recommendation and conclusion may have resulted in an inaccurate answer, for which, up to 13 points of the 36 total would be deducted for this portion of the assignment.

### 2.3. Assessment of Student Perceptions

In order to assess student perceptions of the incorporation of the teaching EMR for the drug information question assignment, a retrospective, pre-post survey instrument was drafted by the authors based on previous research and study objectives. The questions and statements included in the survey were developed to collect student perceptions before and after the assignment in relation to their knowledge and confidence of using the EMR; the importance of EMR access when answering patient-specific questions; and the impact of didactic EMR use on their future performance during clinical rotations, and as a future pharmacist. A draft of survey items was reviewed by four DI faculty members and pilot tested by six reviewers that consisted of student pharmacists, pharmacy residents, and a pharmacy fellow. 

**Figure 1:**
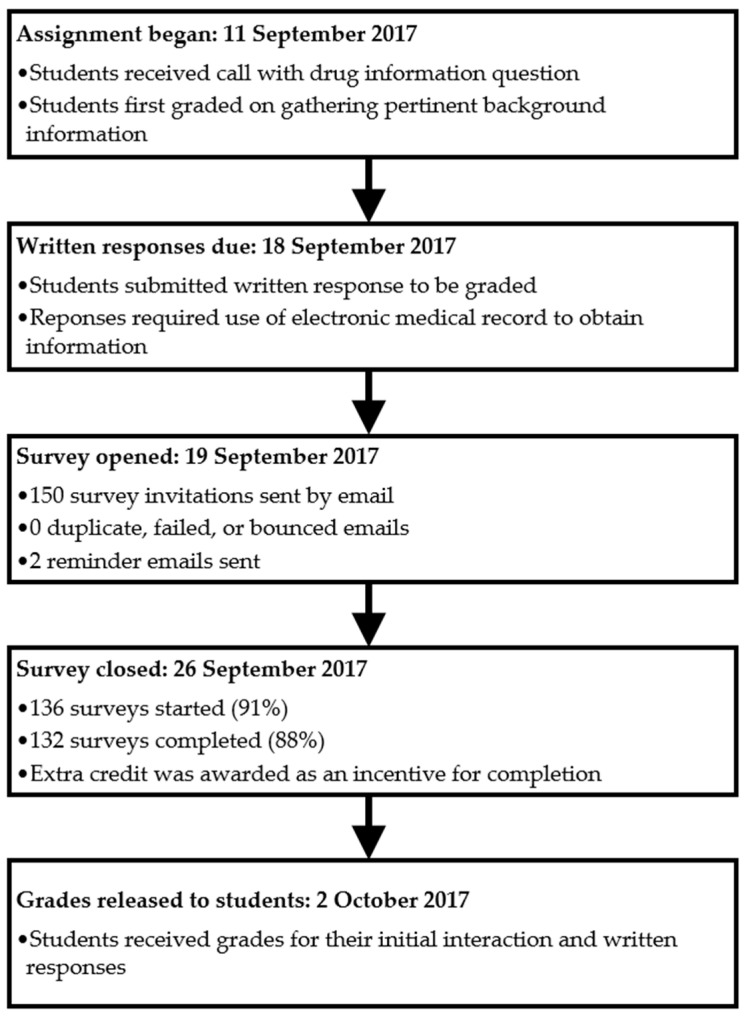
Drug information assignment timeline.

After comments from the survey reviewers were incorporated, the final survey instrument distributed to students contained a total of 23 items. The survey consisted of two main sections. The first section had a series of twelve statements in which participants ranked their initial and current perceptions regarding the use of a teaching EMR for a DI assignment. This was done using a 5-point Likert scale where one equals strongly agree and five equals strongly disagree. The second section, consisting of 11 items, verified that the student had used the EMR as part of their assignment and collected pertinent demographic and background information of the student pharmacist related to EMR usage. The background information included whether or not the student had previous experience with an EMR outside of its use at PUCOP. Students were also provided with the opportunity to enter free text comments. The free text comments were collected for quality improvement purposes for potential future use of an EMR in this course and no formal analyses of these comments were planned. Qualtrics software (Provo, UT, USA) was used to develop and electronically distribute the survey instrument. After completion of the DI assignment, all students enrolled in the course received an electronic invitation to complete the survey instrument. Participation in the survey was voluntary. Students were offered five points extra credit towards their overall course grade and the survey closed prior to the release of grades for the assignment (Figure 1). No student identifiers were collected for the survey. At the end of the survey, students were provided a link to an online form to be able to provide their email to obtain the extra credit. The study was reviewed and granted exempt status by the university’s Institutional Review Board (IRB; Protocol number 1708019615). Informed consent was obtained from all participants and all methods were carried out in accordance with relevant guidelines and regulations.

Data were included for all respondents who completed at least 80% of the survey items. Respondents were allowed to skip questions per local IRB requirements. Data are reported using descriptive statistics where appropriate. Comparisons of initial and current perceptions for each Likert-statement response was analyzed using a Wilcoxon signed rank test. A post-hoc analysis of Likert-statement responses of the subgroups with or without previous EMR experience was also conducted using a Wilcoxon signed rank test to account for this potential confounding variable. Differences between the percentage of respondents who strongly agreed or agreed to each Likert statement compared to those who did not were analyzed using a Chi square analyses. All significance calculations were based on a 95% confidence interval at an alpha of <0.05. Statistical procedures were performed with SPSS Statistics, v24 (SPSS, Inc., Chicago, IL, USA).

## 3. Results

A total of 132 students out of 150 completed the survey instrument, resulting in a response rate of 88%. Participants were mostly female (74%) and had a mean age of 22 ± 1.8 years. Of the respondents, most students (*n* = 35; 26.5%) stated they most likely wanted to work within an inpatient hospital pharmacy upon graduation at that point in time, followed by students wanting to work in an ambulatory care clinic (*n* = 29; 22%). Only 28.8% (38/132) of respondents reported prior experience using an EMR, with 22 of these respondents reporting experience from paid employment. Additional results from respondents of previous EMR use are shown in Table 1.

Almost all survey respondents, 98.5% (130/132) utilized the EMR as part of the DI assignment. Of those, 37.7% (49/130) spent an hour or more accessing the EMR, 35.4% (46/130) spent between 30 min to an hour, 23.1% (30/130) spent under 30 min but over or equal to 15 min, and 3.1% (4/130) spent under 15 min accessing the EMR for the DI question assignment.

Results of the mean and median Likert scale scores for each of the survey items are shown in Table 2. All statements were rated significantly different when comparing the mean Likert scale score for students’ perceptions pre-assignment to students’ perceptions post-assignment (*p* < 0.05). After the assignment, respondents were more likely to strongly agree that access to an EMR is necessary to provide appropriate responses to DI questions in pharmacy practice (mean = 1.44 ± 0.58), and that student pharmacists should be exposed to EMRs during classroom coursework (mean = 1.42 ± 0.67), than before the assignment (mean = 2.02 ± 0.75 and 1.87 ± 0.80, respectively). In contrast, respondents were largely undecided as to whether having access to the EMR would improve their verbal grade (i.e., gathering pertinent background information) for the DI assignment (mean after = 2.93 ± 0.90 vs mean before = 3.05 ± 0.72).

The percentage of respondents who strongly agreed or agreed to each item is presented in Table 2. The largest difference in respondents who strongly agreed or agreed after the assignment compared to those before the assignment was for the statement “I am confident in my ability to look up and obtain patient specific information from an electronic medical record” (20.5%, 82.8% respectively). The second largest difference was for the statement that the EMR used is easy to learn and easy to navigate (21.4%, 51.6%). In contrast, the statement “Access to the teaching EMR for the drug information assignment will improve or has improved my verbal score” had the smallest difference in respondents who strongly agreed or agreed compared to before and after the assignment (18.3%, 29.3%).

A post-hoc analysis of student grades revealed that the average score of the drug information question assignment in 2017 was 52 (±5.26) out of 60 points (86.7%). In 2016, the average score of the drug information question assignment was 50.91 (±4.93) out of 60 points (84.9%).

Within a subgroup analysis of mean Likert-scale scores of students without previous EMR experience, all statements were rated significantly different when comparing students’ perceptions pre-assignment to students’ perceptions post-assignment (*p* < 0.05). For students with reported previous EMR experience, there was a significant difference between mean Likert-scale scores for all statements except for the following: “Access to the teaching EMR for the drug information assignment will improve or has improved my verbal score” (mean after = 3.11 ± 0.91 vs mean before = 3.18 ± 0.77; *p* = 0.763).

The free-text comments submitted by students on the survey were also reviewed post-hoc. There were 44 comments from students out of 132 participants (33.3%) in this section. A common theme from the free-text comments was that it was difficult to look up patient information, especially the patients’ active medication lists. In addition, several students expressed wanting to have had more training or tutorials of the EMR in addition to what they had already received prior to this assignment. 

## 4. Discussion

This study evaluated the incorporation of a teaching EMR within didactic pharmacy curricula by having student pharmacists answer patient-specific drug-related questions. Through this exercise, the importance of patient-centered care was emphasized within a drug information course and students had improved perceptions of their knowledge and confidence regarding the use of an EMR within pharmacy practice by being able to evaluate a patient’s medication history, history of present illness, and other aspects of the medical chart that were needed to be able to answer the DI question. This was demonstrated by the improvements seen in the mean Likert scale scores within the survey instrument. 

It is important for students to understand the expectation of becoming “practice-ready” with health informatics tools, including EMRs, upon graduation, as well as ensuring they are better prepared for future introductory and advanced pharmacy clinical practice experiences. Frequent and early exposure to different aspects of health informatics may help to better prepare these students. Through the implementation of an EMR as part of a DI question assignment early within their didactic courses, student perceptions of needing to be “practice-ready” increased, as shown through the survey results.

In terms of students’ knowledge, while there was no formal analysis of learning outcomes associated with the implementation of the EMR, the slight increase in the average of the drug information question assignment scores from 2016 to 2017 suggests a potential association between perceived benefit and learning outcomes. This potential association aligns with motivation theories in learning, including the self-determination theory [17,19].

In this study, it was also found that overall student perceptions on EMRs had improved after the DI question assignment, regardless of previous exposure to an EMR outside PUCOP. This was demonstrated in that most statements had a significant difference in average Likert-scale scores before and after the assignment. 

As this assignment was done outside of a laboratory session, a measure that was collected within the survey was an estimate by the students in how long they used the EMR. Most students had spent an hour or more using the tool, which may have impacted their perceptions of their confidence and knowledge and their performance on the assignment. Future studies may help better inform the out-of-class time required and may help identify any correlations on student perceptions and outcomes. Previous studies that utilized an EMR within a laboratory course, had done so over at least one 3-h laboratory class session [9,12]. The use of an EMR in the current setting is relatively comparable for the participants that had used the EMR for an hour or more. 

Previous studies have analyzed different approaches of incorporating health informatics topics and found that having different types of tools in didactic courses helped to improve students’ confidence and knowledge [8,9,10,11]. For example, in Hincapie et al., a pharmacy informatics module was incorporated within a semester long DI course [9]. This module utilized online videos, individual and team readiness assurance tests, and team-based learning discussion to educate students on pharmacy health informatics topics. This approach found student confidence in health informatics had increased based off of student survey scores [9]. Frenzel et al. developed an EMR within a pharmaceutical care laboratory with patient specific case studies [8]. Students in this course developed patient care recommendations via creation of SOAP (i.e., subjective, objective, plan, and assessment) notes within the EMR on specific therapeutics topics. This study found an improvement in students’ skills of disease state management in patient-centered care [8]. 

To our knowledge, this is the first study specifically examining incorporation of an EMR within a drug-related question assignment outside of a laboratory course. In having another avenue within didactic instruction for student pharmacists to gain exposure, students can further build on their knowledge and confidence of health informatics prior to entry into pharmacy practice. Incorporating use of a teaching EMR pairs well within a DI and literature evaluation course where students are provided instruction on strategies to answer drug-related questions. An important area of emphasis in our course is the collection of pertinent background information to fully understand the context of the question. Without proper context and patient-specific background information, responses provided to drug-related questions may be too general and not adequately address the true need from the requestor. This can potentially compromise the ability of the pharmacist to provide effective patient care. 

Previous literature has shown that failure to collect information regarding the patient-specific context of an inquiry can reduce the quality of responses provided by pharmacists [20]. Calis et al. tested the quality of responses by asking specific drug-related questions to DI centers across the United States [20]. One case example requested the following, “I would like to try erythromycin for one of my patients who has diabetic gastroparesis” [20]. This request required DI centers to identify that the patient had not received metoclopramide and had no contraindications for metoclopramide [20]. As per current clinical practice guidelines and standard of care at the time of this study on the management of gastroparesis, metoclopramide is considered first line and therefore the final recommendation to the requestor should include this information for a complete and accurate response [20,21].

The American Society of Health-System Pharmacists have also established guidelines for pharmacists providing drug information [22]. Within these guidelines, obtaining complete background information, i.e., examining medical record data, is part of a systematic approach to responding to DI requests [22]. Use of the teaching EMR to connect patient cases to DI questions provides students with a realistic example of the importance of incorporating the patient-specific context during the process of providing DI.

Student respondents had the option of providing free text responses within the survey instrument. Some of the comments received are aligned with the results. For example, one student stated that “It was very handy and it will give us more experience prior to our rotations.” Another noted that it was helpful to look up a patient’s information. However, several students noted that the EMR was difficult to navigate or difficult to determine active problems and medications for a patient. Given this feedback, students’ knowledge and confidence scores could potentially have been influenced by the EMR’s ease of use. On average though, student respondents had agreed that the EMR used at the PUCOP is easy to learn and easy to navigate based on Likert-scale scores after the assignment. 

This specific assignment was the first exposure to an EMR for most students within this course. Given this information, incorporating an EMR early within didactic instruction may positively impact students’ perceptions of health informatics tools prior to entry into pharmacy practice, as seen by the survey instrument results. 

This study is limited in that we relied on the students’ perceptions of their improvement in knowledge of an EMR. While there was consideration to incorporate the students’ scores of the DI assignment as part of the study, covariates were identified (i.e., differences in the DI question assigned or overall academic performance of individual students) at the time the study was conducted. In addition, several theories on motivations in learning have linked confidence with motivation. In self-determination theory, which consists of the components of autonomy, competency, and relatedness, the perception of being knowledgeable about a topic plays a role in an individual’s competence and subsequently their motivation [19]. Given the positive perceptions that students had with the EMR for this assignment, future studies may be then conducted to evaluate student learning outcomes and to validate this theory. 

An additional limitation is that this survey was done utilizing a retrospective pre-post design. This design was chosen to prevent response-shift bias because the majority of students had no prior experience using an EMR. That is to say, if students had no prior exposure to EMR use, then they may not be able to accurately assess their pre-activity perceptions of knowledge and confidence. However, this could lead to social desirability bias, cognitive dissonance, or memory bias from the respondent. Although these biases exist, the improvements that were found in student perceptions are still impactful and provide insight into how an EMR may be utilized with a drug information assignment outside of a laboratory course. Our study also provides insight for future studies where alternative forms of evaluation may be utilized. Finally, there is a limitation in that the survey instrument focused on short-term student perceptions. Additional research that assesses student pharmacist perceptions in the long term, including after clinical rotations, and entry into pharmacy practice, is warranted to better understand the impact on integration of health informatics tools early within didactic instruction. 

## 5. Conclusions

Implementation of an EMR within didactic instruction may serve as the first exposure to health informatics tools for students and may also positively impact students’ perceptions on these tools prior to entry into pharmacy practice. A patient-specific DI assignment is an opportunity to incorporate use of an EMR early within didactic instruction for student pharmacists and emphasizes the importance of patient-centered care.

## Figures and Tables

**Table 1:** Additional characteristics of respondents with previous EMR experience.

	Respondents *n* = 38, *n* (%)
Previous exposure to EMR practice setting ^a^	
Hospital	19 (50)
Ambulatory Care Office	3 (7.9)
Academic	1 (2.6)
Community Based System	20 (52.6)
Managed Care Pharmacy	1 (2.6)
Prefer not to say	1 (2.6)
Previous exposure to EMR practice type ^a^	
Paid job or internship outside of IPPE	22 (57.9)
IPPE	20 (52.6)
Academic	3 (7.9)
Volunteer work outside of IPPE	3 (7.9)
Type of EMRs exposure ^a^	
Cerner	11 (28.9)
Epic	5 (13.2)
Allscripts (Sunrise Clinical Manager)	3 (7.9)
NextGen	1 (2.6)
Athena	1 (2.6)
eClinicalWorks	2 (5.3)
McKesson	4 (10.5)
Others, not specified	5 (13.2)
Unknown	9 (23.7)
Approximate length of time working with EMRs	
<2 weeks	5 (13.2)
≥2 weeks, but <1 month	3 (7.9)
≥1 month, but <1 year	18 (47.4)
≥1 year	11 (28.9)
Prefer not to say	1 (2.6)

EMR, electronic medical record; IPPE, introductory pharmacy practice experience. ^a^ Respondents were allowed to select more than one answer. Note: Only respondents who answered ‘Yes’ to previous exposure to EMRs were prompted to complete this portion of the survey.

**Table 2:** Student Pharmacists’ Perceptions of Use of an EMR for a Drug Information Question Assignment.

Statement	Pre Assignment		Post Assignment		
	Mean (SD) Median	Agreed or Strongly Agreed, *n* (%)	Mean (SD) Median	Agreed or Strongly Agreed, *n* (%)	*p* Value ^a^
I am confident in my ability to look up and obtain patient specific information from an electronic medical record (EMR). (*n* = 132, 128)	3.49 (1.04) 4	27 (20.5)	2.09 (0.70) 2	106 (82.8)	<0.001
Access to an EMR is useful for completing the drug information question assignment. (*n* = 132, 131)	2.08 (0.78) 2	95 (71.9)	1.63 (0.81) 1	120 (91.6)	<0.001
Access to patient information in an EMR is necessary to provide appropriate responses to drug information questions in pharmacy practice. (*n* = 132, 131)	2.02 (0.75) 2	102 (77.2)	1.44 (0.58) 1	130 (99.3)	<0.001
Using the teaching EMR to complete the drug information assignment will improve or has improved my knowledge of EMR systems. (*n* = 132, 130)	2.30 (0.79) 2	81 (61.4)	1.70 (0.80) 2	116 (89.3)	<0.001
Using the teaching EMR to complete the drug information assignment will improve or has improved my confidence in using EMR systems. (*n* = 131, 130)	2.34 (0.76) 2	76 (58.0)	1.84 (0.81) 2	110 (84.7)	<0.001
Student pharmacists should be exposed to EMRs during classroom coursework. (*n* = 131, 131)	1.87 (0.80) 2	105 (80.2)	1.42 (0.67) 1	127 (96.9)	<0.001
The teaching EMR used is easy to learn and easy to navigate. (*n* = 131, 130)	3.09 (0.84) 3	28 (21.4)	2.60 (0.99) 2	67 (51.6)	<0.001
The teaching EMR used to complete the drug information assignment will improve my performance during upcoming IPPE and APPE rotations. (*n* = 132, 131)	2.33 (0.74) 2	78 (59.1)	1.98 (0.78) 2	104 (79.4)	<0.001
Access to the teaching EMR for the drug information assignment will improve or has improved my verbal score. (*n* = 131, 130)	3.05 (0.72) 3	24 (18.3)	2.93 (0.90) 3	38 (29.3)	0.03
Access to the teaching EMR for the drug information assignment will improve or has improved my written score. (*n* = 132, 131)	4.71 (3.08) 2	67 (50.8)	3.05 (2.70) 2	99 (75.5)	<0.001
The integration of the teaching EMR within the drug information course will enhance or has enhanced my overall learning experience. (*n* = 132, 131)	2.23 (0.75) 2	88 (66.7)	1.84 (0.73) 2	114 (87.1)	<0.001
It is an expectation that pharmacy graduates are ready to appropriately use health information tools, including EMRs. (*n* = 132, 130)	1.95 (0.79) 2	103 (78.0)	1.62 (0.80) 1	116 (89.3)	<0.001

APPE, advanced pharmacy practice experience; EMR, electronic medical record; IPPE, introductory pharmacy practice experience; SD, standard deviation. ^a^ *p* values were calculated using the comparisons of initial and post-assignment mean scores for each Likert-statement response with a Wilcoxon signed-rank test. Note: Responses based on a five-point Likert scale (1 = strongly agree, 2 = agree, 3 = undecided, 4 = disagree, 5 = strongly disagree). Students were allowed to skip questions within the survey. Results were analyzed using the total of responses recorded for each Likert statement, with the first *n* value reported representing the total number of participants that selected a choice for the pre assignment Likert-statement, and the second *n* value representing the total number of participants that selected a choice for the post statement.

## Data Availability

The data presented in this study are available on request from the corresponding author.

## Appendix A

### Example Drug Information Question Given to Students by an Instructor

Initial prompt: “Hi this is Dr. [name of instructor], I need to know the starting dose of naltrexone”.

Background: This requestor is a primary care physician who would like to prescribe naltrexone for a patient. He/she is not familiar with the correct dose.

Patient-Specific Information: *

Demographics:

• Name: Faye Dugan
• MRN: 3938703891, 45 year old female
• Height/Weight: 5 feet 7 inches; 136 pounds

HPI: The patient presented to the physician’s office for follow-up on depression and suicide ideation. Patient admits that she is afraid she will not be able to pay for her office visit as she has recently lost a large amount of money gambling. Upon further evaluation, patient admits to going to casinos multiple times a week, and had recently been fired from work due to missed days. While the patient no longer has suicidal thoughts or feels depressed, she often gets “extreme cravings” which are only relieved by gambling. The doctor would like to know if naltrexone can be used to treat pathological gambling, and if so, what is the recommended dose for this specific patient.

PMH: Low back pain; major depression; suicidal thoughts; alcohol abuse; anxiety

Allergies: “I don’t know” (student would need to locate in EMR)

Current Medications: buspirone, cyclobenzaprine, prescription strength ibuprofen, prescription strength naproxen, fluoxetine (student would need to locate dosing regimen for each in EMR)

OTC Medications: “I don’t know” (student would need to locate in EMR)

Social History: Tobacco (−), alcohol (+), illicit drug use (−)

Available Vital Signs and Lab Results:

• Temperature: 98.9 degrees Fahrenheit
• Blood Pressure: 132/62 mmHg
• Oxygen Saturation: 99%
• Pulse: 88 bpm
• Respiratory Rate: 19 breathes per minute
• Pain Score: 2
• Renal Function Labs: “I don’t know” (student would need to locate in EMR)
• Liver Function Labs: “I don’t know” (student would need to locate in EMR)
• Required Background Questions for Students to Ask for Full Credit:
• What is the indication for naltrexone? (pathological gambling)
• What is the patient’s age and weight? (age as above, student to locate weight in EMR)
• What is the status of the patient’s renal and hepatic function? “I don’t know” (in EMR)
• What are the patient’s current medical problems? (provide PMH as above)
• What other medications is the patient taking right now? (as above)
• What are the doses of the patient’s current medications? “I don’t know” (in EMR)
• Does the patient take any OTC or herbal medications? “I don’t know” (in EMR)
• Does the patient have any allergies? “I don’t know” (in EMR)

Abbreviations: bpm, beats per minute; EMR, electronic medical record; HPI, history of present illness; MRN, medical record number; OTC, over-the-counter; PMH, past medical history

* Patient-specific information listed are false identifiers that had been built for the purpose of this study
